# Neurovascular coupling (NVC) in newborns using processed EEG versus amplitude-EEG

**DOI:** 10.1038/s41598-021-88849-6

**Published:** 2021-05-03

**Authors:** Yudhajit Das, Xinlong Wang, Srinivas Kota, Rong Zhang, Hanli Liu, Lina F. Chalak

**Affiliations:** 1 Department of Bioengineering, University of Texas at Arlington, Arlington, TX USA; 2 Department of Neurological Surgery, University of Texas Southwestern Medical Center, Dallas, TX USA; 3 Departments of Neurology and Internal Medicine, University of Texas Southwestern Medical Center, Dallas, TX USA; 4 Department of Pediatrics, University of Texas Southwestern Medical Center, Dallas, TX USA

**Keywords:** Encephalopathy, Hypoxic-ischaemic encephalopathy, Neonatal brain damage

## Abstract

There is a critical need for development of real time physiological biomarkers for birth asphyxia that constitutes a major global public health burden. Our recent study (Scientific Reports, *V10:9183, 2020*) established a novel non-invasive neurovascular coupling (NVC) assessment in newborns using dynamic wavelet transform coherence (WTC) analysis irrespective of different aEEG algorithms. As an extended study, the current paper examines whether the variability in processed EEG and amplitude-EEG (aEEG) outputs would impact the determination of NVC in newborns with encephalopathy. Concurrent processed EEG tracings and regional near infrared spectroscopy (NIRS)-based cerebral tissue oxygen saturation (SctO2) readings during a period of twenty hours in their first day of life were selected and processed in this study. After bandpass-filtered in 2–15 Hz, rectified, and down-sampled at 0.21 Hz, the processed EEG tracings along with NIRS-SctO2 (0.21 Hz) were used to perform WTC analysis, followed by comparison of WTC-metrics between SctO2-processed EEG coherence and SctO2-aEEG coherence using Bland–Altman statistics. Our results demonstrated high and significant correlation (R2 = 0.96, p < 0.001) between NVC assessments by SctO2-processed EEG and SctO2-aEEG coherence, confirming that band-passed, rectified, and down-sampled processed EEG, or aEEG, can be paired with NIRS-SctO2 to assess NVC in newborns with encephalopathy. Findings indicate the feasibility of a simpler approach to NVC in neonates by using directly processed EEG, instead of aEEG.

## Introduction

Asphyxia impairs fetal cerebral blood flow and is manifested postnatally by neonatal encephalopathy (NE) using the clinical Sarnat stages. A real clinical challenge has been the difficulty to clinically discern the encephalopathy severity within the short therapeutic window to guide decision making regarding the initiation of hypothermia. There is a critical need for development of real time physiological biomarkers for birth asphyxia that constitutes a major global public health burden. Brain function monitoring using amplitude integrated-EEG (aEEG) or EEG is recommended as a standard of care practice in neonatal hypoxic-ischemic encephalopathy (HIE)^1–4^. In 2017, we first established a new neurovascular wavelet bundle methodology which enables an unprecedented real time analysis of neurovascular coupling non-invasively at the bedside^5^. In a subsequent study in 2020, we have demonstrated the robustness of neurovascular coupling (NVC) based on wavelet transform coherence (WTC) analysis between various aEEG signal and SctO2 in newborns with encephalopathy irrespective of processing algorithms variability in aEEGs^6^.

Different EEG and aEEG devices are being used at the bedside in clinical practice and increasingly used in the neonatal intensive care unit (NICU) for infants with hypoxic-ischemic encephalopathy (HIE), which introduce possible heterogeneity of practice, complicate the analysis of results, and affect patient inclusions in future trials. The current paper demonstrates a simpler and more direct approach to measure NVC in neonates by using processed EEG, instead of amplitude-EEG (aEEG). The procedure to obtain processed EEG included three simple steps: (1) bandpass-filtering in 2–15 Hz, (2) rectification, and (3) down-sampling at 0.21 Hz to match with the sampling rate of SctO2^6^. This approach may potentially expand or enable a conventional EEG system to simultaneously display continuous EEG (cEEG) and NVC by using processed EEG in real time. The impact of different processing methods of EEG with hybrid devices on the novel WTC-based NVC analysis is not known.

Specifically, we now examine the rigor and consistency of the novel NVC methodology using processed EEG tracings directly from EEG hybrid or from AEEG machines to measure NVC assessment obtained from infants with asphyxia monitored in the first day of life, aiming to compare WTC-metrics between SctO2-processed EEG coherence and SctO2-aEEG coherence using Bland–Altman statistics, and evaluate agreement between two the NVC assessment tools.

## Results

**Figure 1:**
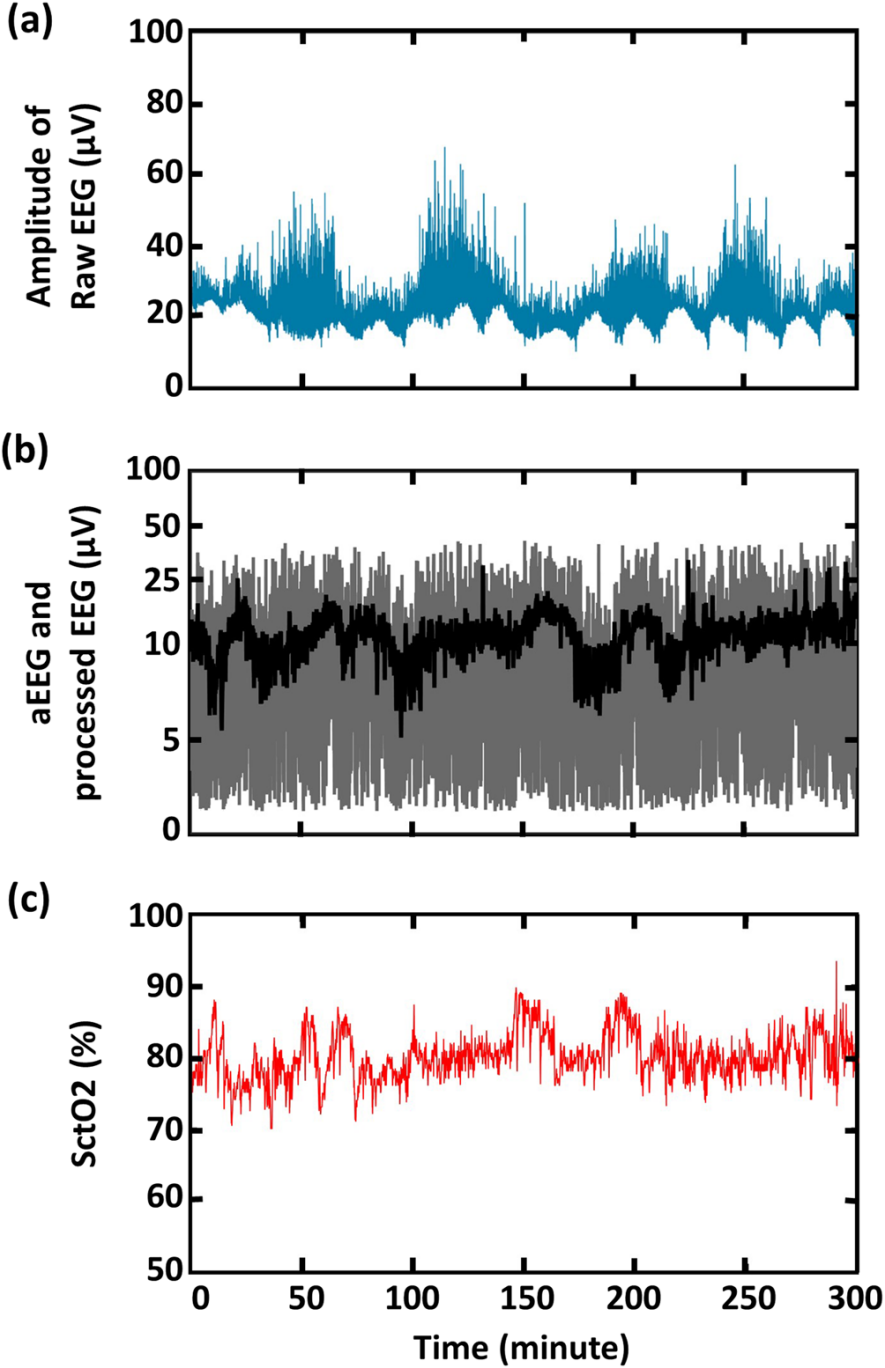
(**a**) An example of amplitude tracing of 5-h raw EEG recorded with a 256-Hz sampling. The y-axis represents amplitude of raw EEG in μV, plotted with a linear scale, while x-axis represents time in minute. (**b**) Three-step processed EEG tracing (processed EEG, gray trace) plotted together with aEEG (black trace) derived with WU-NEAT^7^. The y-axis represents amplitude of aEEG or processed EEG in μV, plotted with a linear scale between 0–10 μV and a log scale for any value larger than 10 μV. (**c**) The corresponding SctO2 signal.

As an example, Fig. 1a shows a temporal trace of 5-h, 256-Hz raw EEG signal, after bandpass filtering (0.1–100 Hz) and rectification, from a normal reference control neonate (Neonate #1). Figure 1b shows an aEEG tracing derived using WU-NEAT method (black trace)^7^ together with a three-step processed EEG (gray trace), both derived from the original EEG signal (i.e., Fig. 1a). This example illustrates that even though both output signals were derived from the same raw EEG tracing, the variation between processed EEG and aEEG tracings is visually clear. We extracted a 5-h time segment to clearly examine the differences among the two EEG curves (aEEG and processed EEG), visually plotted together in Fig. 1b. The corresponding SctO2 signal recorded simultaneously with EEG in Fig. 1c for easy comparison.

### Effect of SctO2-aEEG and SctO2-processed EEG on WTC-derived NVC

**Figure 2:**
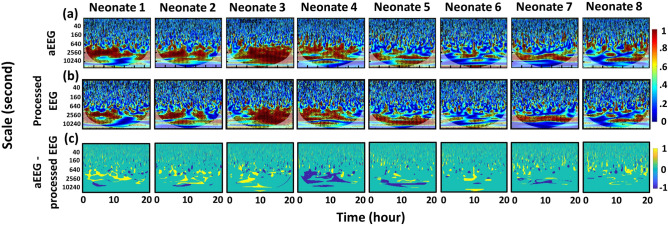
Time-scale coherence maps of NVC in the selected cohort. The x-axis represents time in hours, the y-axis represents scale in seconds, and the color scale represents the amplitude of WTC coherence, R^2^. The areas with significant NVC (p < 0.05) are contoured with black lines and filled by red color in each panel. Top and bottom rows correspond to WTC-based SctO2-aEEG and SctO2-processed EEG coherence (R^2^), R_SctO2→aEEG_ and R_SctO2→processed EEG_, for each case (Neonate #: N1-N8). For each panel of the top row, aEEG tracings were derived using WU-NEAT method^7^; for each panel of the bottom row, processed EEG tracings were obtained by three-step preprocessing means: bandpass filtering (2–15 Hz), rectifying, and down sampling (0.21 Hz) on processed EEG tracings.

Following the same method as published before^5,6^, we obtained and plotted WTC-based time-scale coherence maps based on both SctO2-aEEG and SctO2-processed EEG coherence as shown in Fig. 2a,b, respectively, taken from each of the 8 neonates. Each column represents the coherence patterns per neonate, exhibiting relatively comparable and consistent time-scale WTC map patterns in the scale range of 640–10,240 s (0.1–1.6 MHz) despite the large difference in data processing procedures to obtain NVC. To better quantify time-scale coherence difference between the two calculation methods, Fig. 2c presents difference maps by subtracting the maps with the processed EEG from those with aEEG for each respective neonate, demonstrating the areas where time-scale coherence differed by the two methods. It is seen that the aEEG approach resulted in apparently more coherence areas (yellow regions) in time-scale maps than those (blue regions) by the processed EEG method in most of neonates.

### Comparison of total number of pixels within statistically significant contours across scale

**Figure 3:**
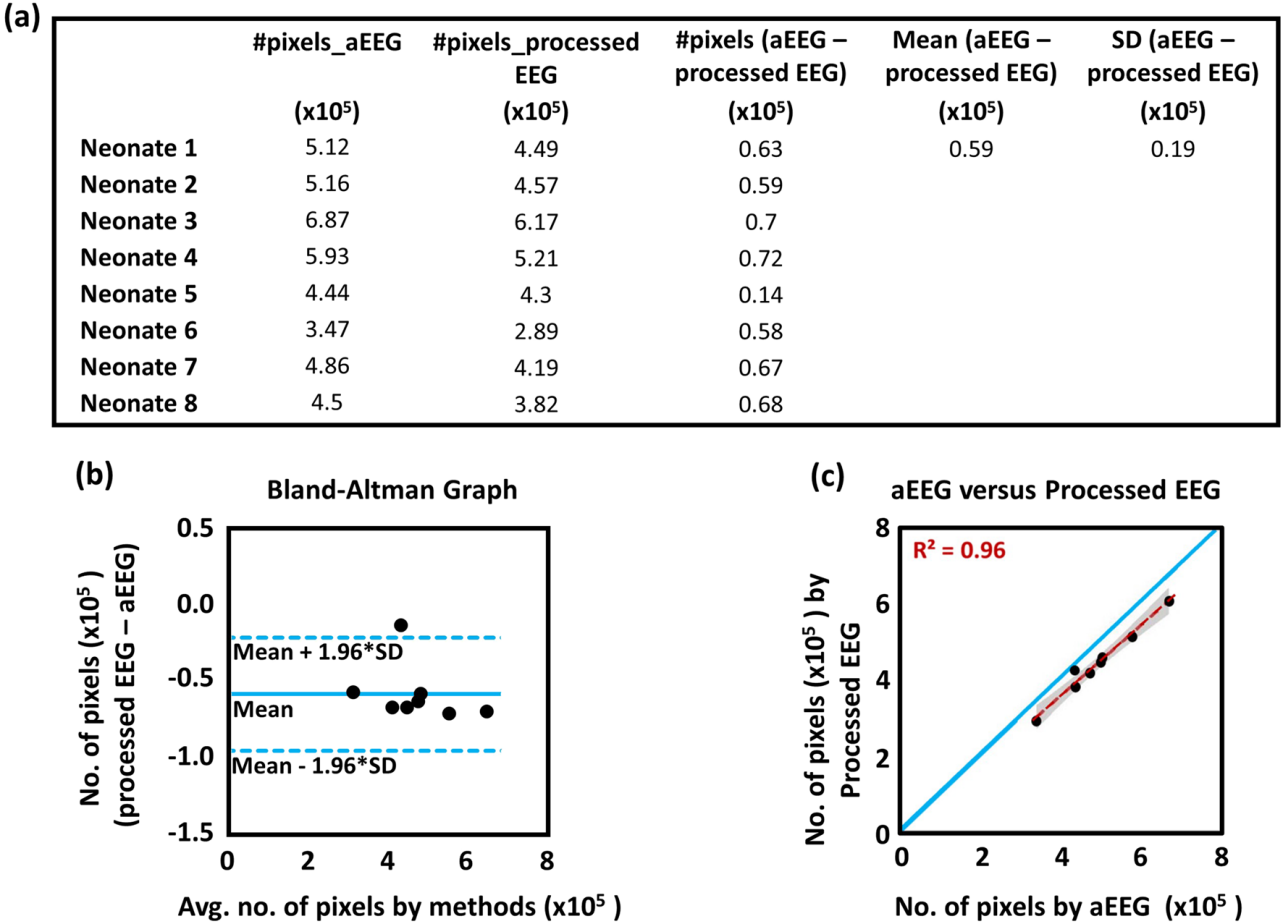
(**a**) NVC metrics showing significant coherence pixels derived with aEEG and processed EEG tracings for each neonate, the difference between the two types of total pixel numbers per neonate, mean and standard deviation (SD) of the difference in NVC across the entire cohort. (**b**) Bland–Altman plot to show WTC-derived agreements using processed EEG versus aEEG temporal tracings. The horizontal upper and lower blue lines mark the limits of agreement, defined as the mean difference ± 1.96 SD (i.e., within 95% confidence level). (**c**) A comparison of total numbers of significant coherence pixels derived using the two methods. A blue “line of identity” demonstrates the perfect match between the results from the two respective methods. The red line in the panel shows the linear regression. The linear regression line has a slope of 0.93 (95% confidence interval: 0.76 to 1.09) and an intercept of − 0.21 (95% confidence interval: − 1.08 to 0.65). Pearson’s correlation coefficient between the two methods is R = 0.984 with 95% confidence interval [0.910, 0.997], p < 0.001. The gray shaded region represents the 95% confidence interval.

Figure 3a shows the total numbers of pixels for NVC, that are significantly coherent based on aEEG and processed EEG for each newborn. To compare the results in NVC derived from the two methods, we also calculated the difference between the two types of total pixel numbers, the mean and standard deviation (SD) of the difference in NVC across the entire cohort, as listed in Fig. 3a. Furthermore, Fig. 3b shows a Bland–Altman (BA) plot to compare the NVC values derived by the two methods, demonstrating a slight negative bias offset in NVC derived from the processed EEG as compared with that from aEEG. However, Fig. 3c shows a highly significant correlation (R^2^ = 0.96; p < 0.001) between the NVC metrics determined by the two methods.

## Discussion

Key finding in the current study is that WTC-based NVC assessment can be performed by quantifying the NVC coherence using either directly processed EEG or aEEG machines ensuring the rigor of this analysis with variable devices in newborns with encephalopathy. This approach eliminates several unspecific data processing pipelines when using commercial devices and accommodates the large variability in practice as some centers use EEG, while others use amplitude EEG in the clinical protocols of monitoring HIE.

Multimodal neuro-monitoring (e.g., EEG and aEEG) to assess brain function and cerebral oxygenation delivery (NIRS) promises to provide estimates of neurovascular functions in new neuroprotective trials to target newborns with encephalopathy at higher risk who need added interventions. There is a long history of EEG as a tool for predicting outcomes after hypoxia ischemia and other neurologic conditions^8,9^. Different aEEG and EEG devices are currently being used at the bedside in clinical practice and increasingly being used in NICU in the encephalopathic newborns. Such devices include new digital EEG systems that can display simultaneous cEEG and processed EEG in real time. Currently those hybrid EEG/processed EEGs as in this investigation are best able to predict good and poor outcomes after hypoxic ischemic injury by providing optimal detection of seizures per the Standard American Clinical Neurophysiology Society recomendations^10,11^.

We have previously reported the rigor and robustness of the WTC analysis to quantify NVC enabling an unprecedented real time analysis of NVC non-invasively at the bedside using SctO2 and aEEG^6^. aEEG has traditionally been applied to surveil the brain development and screen cerebral pathologies by evaluating the background pattern and sleep-awake cycles in neonatal encephalopathy^12^. The digital calculation of aEEG was first described by Maynard et al.^13^. Many studies have been thereafter published using normal and abnormal amplitude margin ranges for preterm and term infants^4,14,15^. These output amplitude tracings are displayed in a semilogarithmic (linear 0–10 μV, logarithmic 10–100 μV) and time-compressed (6 cm/h) format^13^, following some general procedure of aEEG data processing which allows clinicians to get an overall idea of the predominant background pattern and changes at the bedside.

The multitude of differences among aEEG algorithms have been discussed in our previous study; careful selection of the algorithm settings was recommended at the bedside, as these differences could potentially impact the clinical decision makings by the clinicians^6,16^. Caution is needed as differences in preprocessing can affect both the upper and the lower border of the tracing^17,18^. In contrast, the current study shown in Fig. 3 demonstrates that the WTC-based NVC assessment is highly correlated with that when the processed EEG tracings are used after three simple processing steps to match the NIRS signal. This adds to the versatility of the NVC analysis by confirming that it can be quantified using the wavelet analysis with a processed EEG or aEEG depending on what is utilized for clinical practice.

Technically, it is noted that amplitudes of raw EEG signal varied in a large range between ~ 10 and 60 μV within hours as shown in Fig. 1a. Thus, it is necessary to perform multiple signal processing steps in order to remove large noise and obtain smoother EEG tracings for NVC quantification. In Fig. 1b, we demonstrate that the bandwidth of the margin amplitudes of processed EEG tracings was broader than that of the aEEG tracings. This is likely stemming from the simple bandpass filter in processed EEG that ranges from 2 to 15 Hz. This contrasts to the aEEG tracings that have been through multiple and variable data smoothing, rectification, filtering (some of which are undisclosed in commercial devices). All these steps have contributed to a narrower aEEG bandwidth compared to that of the processed EEG.

The results given in this study have two aspects. First, processed EEG can be used to quantify the WTC-based NVC in newborns since the results are highly and significantly correlated with those given by aEEG. Second, there exists a constant bias/offset in the NVC assessment by processed EEG with respect to that by aEEG. This offset can be possibly attributed to fewer processing steps in processed EEG, which resulted in a broader amplitude range of the resultant EEG output signal. On the other hand, aEEG after performing numerous processing steps resulted in a comparatively much narrower or smaller amplitude range as visualized in Fig. 1. Since WTC used to calculate NVC in this study primarily considers the amplitude shapes of the two input signals when calculating the coherence between them, aEEG signals with less noise and narrower amplitude could potentially demonstrate a slightly higher WTC parameter. However, the offset in processed EEG should have a minimal impact on clinical decision making because the bias can be removed by calibration or taking a ratio-metric comparison of trends for the same newborn.

In conclusion, the key findings in this paper confirmed statistical consistency of WTC analysis to quantify NVC of neonates with HIE using either processed EEG or aEEG tracings. The advantage of using processed EEG in WTC analysis is the complexity reduction in post data processing when using a regular EEG device that can potentially reduce technical burden while keeping the scientific rigor for future translational applications.

## Materials and methods

### Subjects and measurement protocol

In this study, we selected a convenience sample of eight newborns with HIE (same population of infants considered in our prior study^6^) who had a minimum of twenty hours of simultaneous EEG and NIRS-SctO2 monitoring as a standard of care protocol. EEG signals were recorded at a sampling rate of 256 Hz from C3, C4, P3, P4, O1, O2, Cz, and Fz, which were placed on the infant’s scalp according to the 10–20 international system. Obtained electrical signals were then amplified and filtered within a frequency band of 0.1–100 Hz. An INVOS spatially resolved NIRS oximeter, consisting of a neonatal probe with a light emitting diode and two distant sensors, were placed on the infant’s forehead to record SctO2 at a speed of 0.2 Hz. Both EEG and NIRS-SctO2 were interfaced with a multi-device synchronization platform (Moberg Research, Inc., PA, USA) for simultaneous recording of two modalities and then saved for off-line analysis using MATLAB (Mathworks Inc., MA, USA). The time-series information from the cross cerebral electrode pair C3-C4 is used to perform dynamic wavelet coherence analysis with NIRS-SctO2 in all the neonates^6^. The study was approved by the Institutional Review Board of the University of Texas Southwestern Medical Center and informed consent was obtained from parents of each newborn before enrollment.

### Data processing to acquire aEEG

The raw EEG was converted to amplitude-EEG using neonatal EEG analysis toolbox (WU-NEAT) from Washington university, which is an open-source, MATLAB-compatible, clinically validated toolbox to quantify aEEG^7^ and has been described as Method 2 in our prior study^6^.

### Data processing of EEG

**Figure 4:**
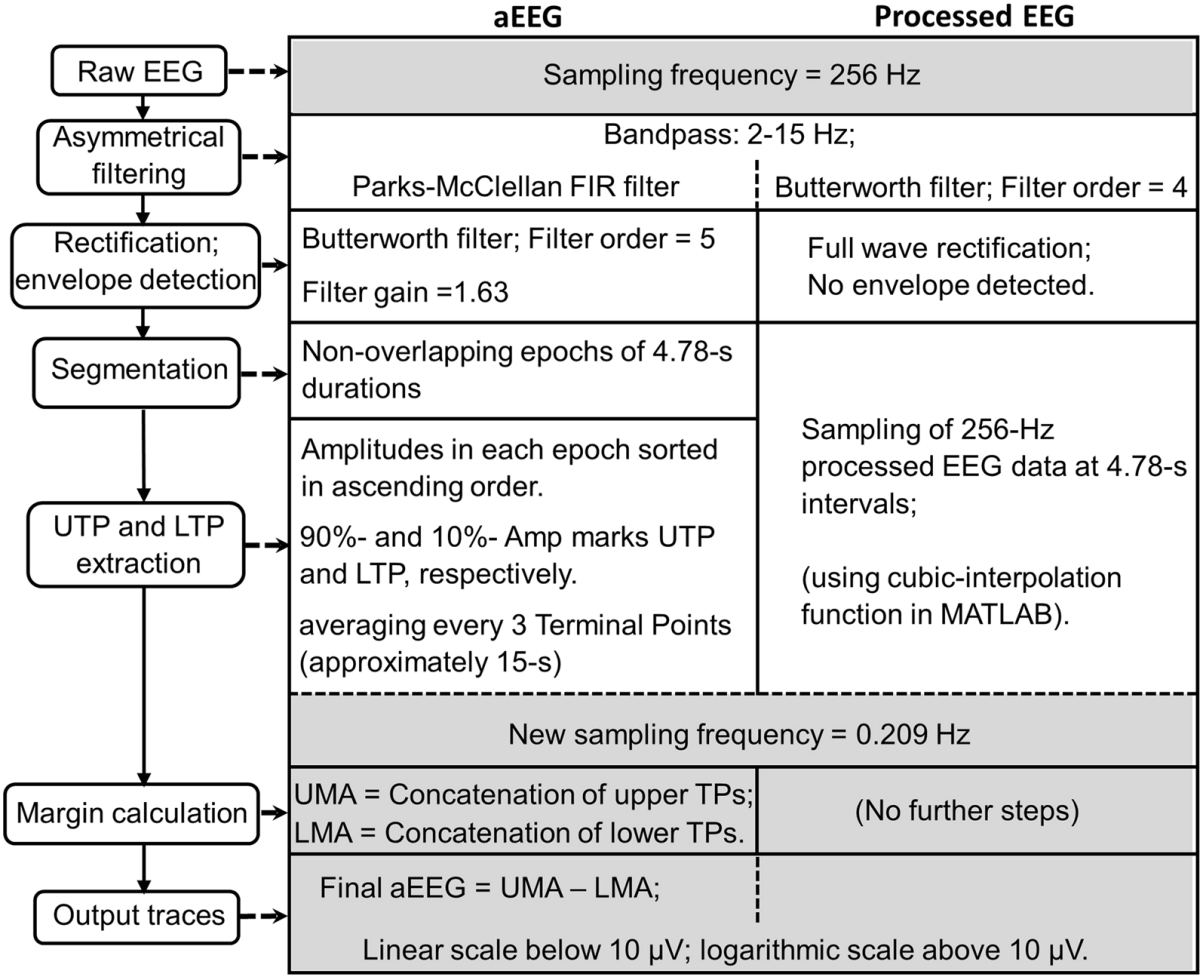
Methodological overview of different steps among two data processing algorithms, enroute to obtaining the common sampling rate for aEEG/processed EEG and NIRS, [Adapted from^6^]; *TPs* terminal positions; *UTP* upper terminal positions; *LTP* lower terminal positions; *UMA* upper margin amplitudes; *LMA* lower margin amplitudes.

The 256-Hz raw-EEG information from C3-C4 channel pair was downloaded from the multi-device synchronization platform (CNS Reader, Moberg Research, Inc., PA, USA). Three steps were performed for processed EEG. First, a 2–15 Hz bandpass filter was applied on the raw EEG signal using a 4^th^ order Butterworth filter. Second, the filtered signal was passed through a full wave rectification. Third, the peak-to-peak amplitudes of the 256-Hz rectified signal were measured and sampled at 4.78-s intervals between a common start and end point using cubic spline interpolation algorithm^19^ to match the dynamics of SctO2 signal (having a sampling rate of 0.21 Hz), which was needed to perform WTC analysis between them. The cubic spline interpolation algorithm allows resampling of the digitized EEG data with minimal distortion to the signal since the interpolation function does not reconstruct artificial spikes and rather assumes the function to be smooth^20^. The processing steps for the output traces derived using two methods are briefly summarized in Fig. 4.

### Quantification of NVC using WTC statistical method

We used a MATLAB-based software package^21^ to perform WTC analysis between the spontaneous oscillations of SctO2 versus processed EEG and of SctO2 versus aEEG signals. WTC is a time–frequency domain analysis, which characterizes the squared cross-wavelet coherence, *R*^2^, against noise background, between two time series at multiple time scales and over time of two pre-specified variables. The two sets of variables selected for the evaluation of WTC-based NVC in this study are SctO2 → aEEG and SctO2 → processed EEG. Details of this method were previously published and can be found in refs. 5 and 6. The thresholding or cut-off criteria in coherence for “significant neurovascular coupling” were provided by the WTC analysis method^21^ that selects time-scale (equivalent to time–frequency) regions having 95% confidence level after performing Monte Carlo simulations. Next, in the time-scale domain, NVC was assessed for each neonate by wavelet metric estimation of total pixel number of significant coherence within all selected regions for both aEEG and processed EEG. Then, the difference between the two types of total pixel numbers per neonate was calculated, followed by determination of the mean and standard deviation (SD) of the difference in NVC across the entire cohort. Statistical analysis to compare agreement between using aEEG and processed EEG tracings in WTC analysis was performed using a Bland Altman curve to detect statistically significant differences in the estimation of their total pixel counts.

### Ethical approval

All methods were carried out in accordance with relevant guidelines and regulations of the institutional review board at the University of Texas Southwestern Medical Center.
